# Sex Bias in Experimental Immune-Mediated, Drug-Induced Liver Injury in BALB/c Mice: Suggested Roles for Tregs, Estrogen, and IL-6

**DOI:** 10.1371/journal.pone.0061186

**Published:** 2013-04-05

**Authors:** Joonhee Cho, Lina Kim, Zhaoxia Li, Noel R. Rose, Monica Vladut Talor, Dolores B. Njoku

**Affiliations:** 1 Department of Anesthesiology and Critical Care Medicine, Johns Hopkins University, Baltimore, Maryland, United States of America; 2 Department of Pathology, Johns Hopkins University, Baltimore, Maryland, United States of America; 3 The W. Harvey Feinstone Department of Molecular Microbiology and Immunology, Johns Hopkins University, Baltimore, Maryland, United States of America; 4 Department of Pediatrics, Johns Hopkins University, Baltimore, Maryland, United States of America; Purdue University, United States of America

## Abstract

**Background and Aims:**

Immune-mediated, drug-induced liver injury (DILI) triggered by drug haptens is more prevalent in women than in men. However, mechanisms responsible for this sex bias are not clear. Immune regulation by CD4+CD25+FoxP3+ regulatory T-cells (Tregs) and 17β-estradiol is crucial in the pathogenesis of sex bias in cancer and autoimmunity. Therefore, we investigated their role in a mouse model of immune-mediated DILI.

**Methods:**

To model DILI, we immunized BALB/c, BALB/cBy, IL-6–deficient, and castrated BALB/c mice with trifluoroacetyl chloride-haptenated liver proteins. We then measured degree of hepatitis, cytokines, antibodies, and Treg and splenocyte function.

**Results:**

BALB/c females developed more severe hepatitis (p<0.01) and produced more pro-inflammatory hepatic cytokines and antibodies (p<0.05) than did males. Castrated males developed more severe hepatitis than did intact males (p<0.001) and females (p<0.05). Splenocytes cultured from female mice exhibited fewer Tregs (p<0.01) and higher IL-1β (p<0.01) and IL-6 (p<0.05) than did those from males. However, Treg function did not differ by sex, as evidenced by absence of sex bias in programmed death receptor-1 and responses to IL-6, anti-IL-10, anti-CD3, and anti-CD28. Diminished hepatitis in IL-6-deficient, anti-IL-6 receptor α-treated, ovariectomized, or male mice; undetectable IL-6 levels in splenocyte supernatants from ovariectomized and male mice; elevated splenic IL-6 and serum estrogen levels in castrated male mice, and IL-6 induction by 17β-estradiol in splenocytes from naïve female mice (p<0.05) suggested that 17β-estradiol may enhance sex bias through IL-6 induction, which subsequently discourages Treg survival. Treg transfer from naïve female mice to those with DILI reduced hepatitis severity and hepatic IL-6.

**Conclusions:**

17β-estradiol and IL-6 may act synergistically to promote sex bias in experimental DILI by reducing Tregs. Modulating Treg numbers may provide a therapeutic approach to DILI.

## Introduction

Drug-induced liver injury (DILI) accounts for more than 50% of all cases of acute liver failure. Halogenated volatile anesthetics, antibiotics, tienilic acid, dihydralazine, carbamazepine, and alcohol induce immune-mediated DILI in susceptible individuals. DILI induced by halogenated volatile anesthetics such as isoflurane, desflurane, or halothane is triggered by neoantigens that are produced when native liver proteins such as cytochrome p450 2E1 (CYP2E1) become covalently modified by trifluroacetyl chloride (TFA) haptens, which are formed by CYP2E1 oxidative anesthetic metabolism [1]–[4]. In susceptible individuals, complex immune responses to these neoantigens induce hepatitis and production of antibodies to native proteins and drug haptens. We have successfully reproduced these mechanisms in a model of anesthetic DILI that utilizes BALB/c mice, which are uniquely susceptible to injury [5].

Experimental anesthetic DILI is characterized by a splenic priming phase that occurs 2 weeks after immunization with S100 liver proteins that are covalently altered by TFA haptens (TFA-S100). Hepatitis develops after 3 weeks and is characterized by the presence of mast cells, neutrophils, and eosinophils [5], cell types that have been reported in infectious and drug-induced hepatitis [6]–[8]. Hepatic injury after 12 weeks confirms that immune responses seen at 2 and 3 weeks initiate injury.

A hallmark of anesthetic DILI is a greater prevalence in women than in men [9]; however, mechanisms responsible for sex bias are not completely characterized. A recent study described how estrogen reduces hepatotoxicity after intraperitoneal exposure to halothane, but the roles of sex steroids in the autoimmune type of this disease have not been investigated [10]. Many autoimmune diseases preferentially affect women. Proposed mechanisms for sex bias involve modulation of T helper cell (Th) 1, Th2, or Th17 pathways by sex steroids [11], [12]. In some studies, estrogen encourages autoreactive B-cell expansion [13]; however, in others, estrogen reduces disease by expanding CD4+CD25+FoxP3+ regulatory T-cells (Tregs) [14].

Tregs belong to a family of regulatory T-cells that inhibit activation, trafficking, and/or effector function of CD4+ and CD8+ T-cells [15], [16] and suppress B-cell and immunoglobulin production [17]. Tregs are deficient and functionally defective in patients with autoimmune hepatitis [18], [19] or fatty liver [20]. However, whether Tregs modulate sex bias in anesthetic immune-mediated DILI is not known.

In the present study, we show that, similar to patients with anesthetic DILI, female BALB/c mice develop more severe experimental DILI than do males. We demonstrate numerical but not functional differences in Tregs. We show that sex bias in the severity of experimental anesthetic DILI is driven by a reduction in the number of Tregs and that this reduction may be induced by pro-inflammatory cytokines such as interleukin (IL)-6 that have been up-regulated by estrogen. To our knowledge, we are the first to uncover critical associations between 17β-estradiol and IL-6 in female BALB/c mice. We also demonstrate therapeutic roles for Tregs in anesthetic DILI sex bias, a finding that could suggest a role for Tregs in therapy for patients who develop immune-mediated DILI from other drugs.

## Materials and Methods

### Materials

Alkaline phosphatase (AKP)-goat anti-mouse IgG was from Millipore (Billerica, MA); AKP-IgG1, IgG2a secondary antibodies, and CYP2E1 supersomes were from BD Biosciences (San Diego, CA); AKP substrate kit was from BioRad (Hercules, CA); anti-IL-10 blocking antibody (rat anti-mouse, clone JES5-2A5) was from Biosource (Camarillo, CA); complete Freund's adjuvant (CFA) was from Difco Bacto (Pittsburgh, PA); β-cyclodextrin (BC), 17β-estradiol (E2), DMEM, hematoxylin and eosin (H&E), and ovalbumin were from Sigma-Aldrich (St. Louis, MO); IL-6 was from PeproTech (Rocky Hill, NJ); IL-6 receptor α (IL-6Rα) blocking antibody (clone D7715A7) was from BioLegend (SanDiego, CA) isotype control (rat IgG1, clone 43414) was from R&D Systems (Minneapolis, MN); minimal essential medium and qualified fetal bovine serum (FCS) were from Invitrogen Corporation (Carlsbad, CA); and pertussis toxin was from List Biologicals (Campbell, CA).

### Mice

Male and female BALB/c ± surgical castration, IL-6-deficient (IL-6−/−), and BALB/cBy mice (8–10 weeks old; The Jackson Laboratory, Bar Harbor, ME) were maintained under pathogen-free conditions at the Johns Hopkins School of Medicine.

### Ethics Statement

This study was accomplished using a protocol approved by the Johns Hopkins Animal Care and Use Committee. (Animal welfare assurance number A3272-01). Johns Hopkins has maintained accreditation by the private Association for the Assessment and Accreditation of Laboratory Animal Care (AAALAC) since 1974. Mouse ovariectomy and castration surgeries were performed by Jackson Laboratories prior to our use.

### Experimental anesthetic DILI

Hepatitis was induced by injecting the mice subcutaneously with TFA-S100 (200 µg) emulsified in an equal volume of CFA on days 0 and 7, as previously described [5]. In addition, 500 ng of pertussis toxin was injected intramuscularly in the hind flank on day 0. In some experiments, mice received anti-mouse/rat IL-6 receptor α (IL-6Rα) antibodies on days 0 and 7. In others mice received anti-rat IL-10 on days 14, 16, 18 and 20. Mice were killed 2 or 3 weeks after the initial immunization. Experiments were carried out 2–3 times (n = 4–5 mice/group).

### Histology

Liver sections (5 µm thick) fixed in 10% neutral buffered formalin and stained with H&E (Histoserv, Gaithersburg, MD) were scored as described [5]: Grade 0, no inflammation or necrosis; Grade 1, minor periportal or lobular inflammation without necrosis; Grade 2, periportal or lobular inflammation involving <50% of the section; Grade 3, periportal or lobular inflammation involving >50% of the section; and Grade 4, inflammation with bridging necrosis.

### Splenocyte isolation

Splenocytes were isolated 2 weeks after initial TFA-S100 immunization as previously described [21] and either stained with the antibody combinations or re-stimulated with media ± CYP2E1 or KLH-TFA (10 µg/mL) and incubated for 72 h at 37°C in 5% CO_2_, 95% air (humidified). After incubation, the cells were pooled by stimulation antigen, counted, washed with PBS/2% FCS, and used in *in vitro* studies.

### Flow Cytometry

Aliquots of 1×10^6^ hepatic lymphomyeloid cells isolated as previously described [5], [22] or splenocytes were incubated for 30 min with FITC/PE/CyChrome™- or APC-labeled antibodies (1∶100): CD4 (L3T4, clone H129.19)/CD8a (Ly-2, clone 53.7.7)/CD3 (clone 145-2C11); CD49bPan-NK cells (clone DX5)/CD45RB220 (clone RA3-6B2)/CD3 (clone 145-2C11); CD11c (clone HL3)/CD8a (Ly-2, clone 53.7.7)/CD3 (clone 145-C11); F4/80 (clone BM8)/Ly-6GGr-1 (clone RB6-8C5)/CD45 (clone 30-F11); CD4 (L3T4, clone H129.19)/CD25 (clone PC61)/FoxP3 (clone FJK-16); PD-1 (CD279, clone J43)/FoxP3 (clone FJK-16)/CD4 (L3T4, clone H129.19); and CD4 (L3T4, clone H129.19)/FoxP3 (clone FJK-16)/CTLA-4 (CD152, clone UC10-489). For FoxP3 detection, splenocytes labeled with FITC-CD4 and APC-CD25 were fixed, permeabilized, and labeled with PE-FoxP3 or with rat IgG2a isotype, which was used as a control (FACScalibur cytometer, CellQuest software [BD BioSciences]). Experiments were carried out in triplicate (n = 4–5/group). All antibodies were from BD Biosciences except for F4/80, PD-1, CTLA-4, and FoxP3, which were from eBioscience (San Diego, CA).

### Antibody ELISA

Antibodies were detected by using S100, CYP2E1, or TFA bound to ovalbumin (TFA-OVA) (5 µg/mL) test antigens and AKP-goat anti-mouse IgG, IgG1, or IgG2a secondary antibodies (1∶1000) [5], [23]. Color development (AKP substrate kit [OD 405 nm]) was measured after 30 min (TFA) or 60 min (S100 and CYP2E1) with an MRX Revelation microplate reader (Dynex Technologies, Revelation software version 4.21). Experiments were carried out in triplicate (n = 4–5 mice/group).

### Cytokine assessments

Cytokines were measured in liver and spleen supernatants (pg/g) obtained from tissues homogenized in DMEM/2% FCS, in splenocyte cultures (pg/mL) [5], [22], or in splenocyte supernatants (pg/mL) from naïve mice treated with E2 (50 nM) or BC (control, 5 mM) using the MILLIPLEX MAP Mouse Cytokine/Chemokine Panel kit, as per kit instructions (Millipore, Billerica, MA). Experiments were carried out in duplicate (n = 5/group).

### *In vitro* assays

Splenocytes cultured in complete media for 72 h in the presence or absence of CYP2E1 or TFA-OVA (10 µg/mL each) [21] were incubated with anti-IL-10 blocking antibody (10 µg/mL), isotype control, or IL-6 (25 ng/mL). BALB/c naïve Tregs were cultured with T effector cells (1∶1), anti-CD3 (5 µg/mL), and anti-CD28 (2.5 µg/mL). T-cell proliferation was measured by ^3^H incorporation [21].

### *In vivo* Treg adoptive transfer

Female BALB/c mice with experimental DILI were injected intravenously with PBS or 1×10^6^ CD4+CD25+Tregs isolated from naïve female BALB/c mice as previously described [20]. Experiments were run in duplicate (n = 4–5group).

### Statistical analysis

Histology scores, cells, antibodies, cytokines, and *in vitro* assays were analyzed between groups by using the Mann-Whitney *U* test or ANOVA with Tukey's post-hoc test (GraphPad® Prism Version 3.02 for Windows, GraphPad® Software, Incorporated, San Diego, CA). A *p* value<0.05 was considered statistically significant.

## Results

### Female BALB/c mice develop more severe experimental, anesthetic-induced, immune-mediated DILI

**Figure 1:**
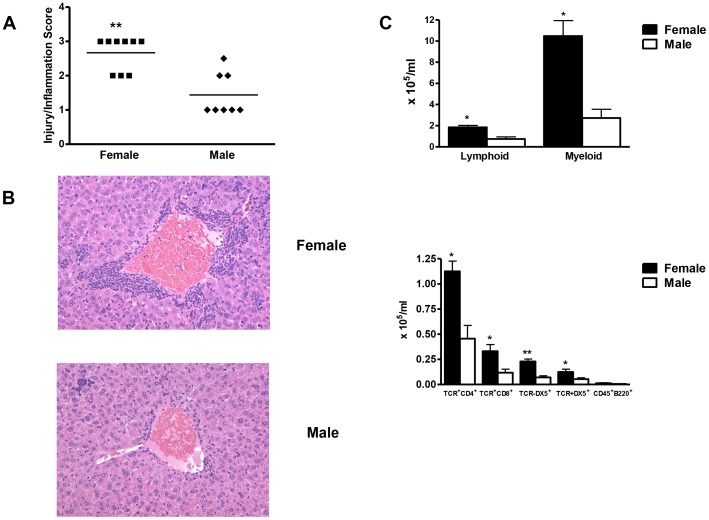
Female BALB/c mice develop more severe experimental anesthetic DILI than do males. (A) Female mice (n = 8) had significantly more severe hepatitis 3 weeks after TFA-S100/CFA immunizations than did males (n = 7/group). (B) Representative liver sections from female and male mice (H&E, magnification 64×). (C) Numbers of hepatic CD4+, CD8+, NK+, and NKT+ cells were significantly higher in females than in males. mean ± SEM. *p<0.05, **p<0.01, ***p<0.001.

**Figure 2:**
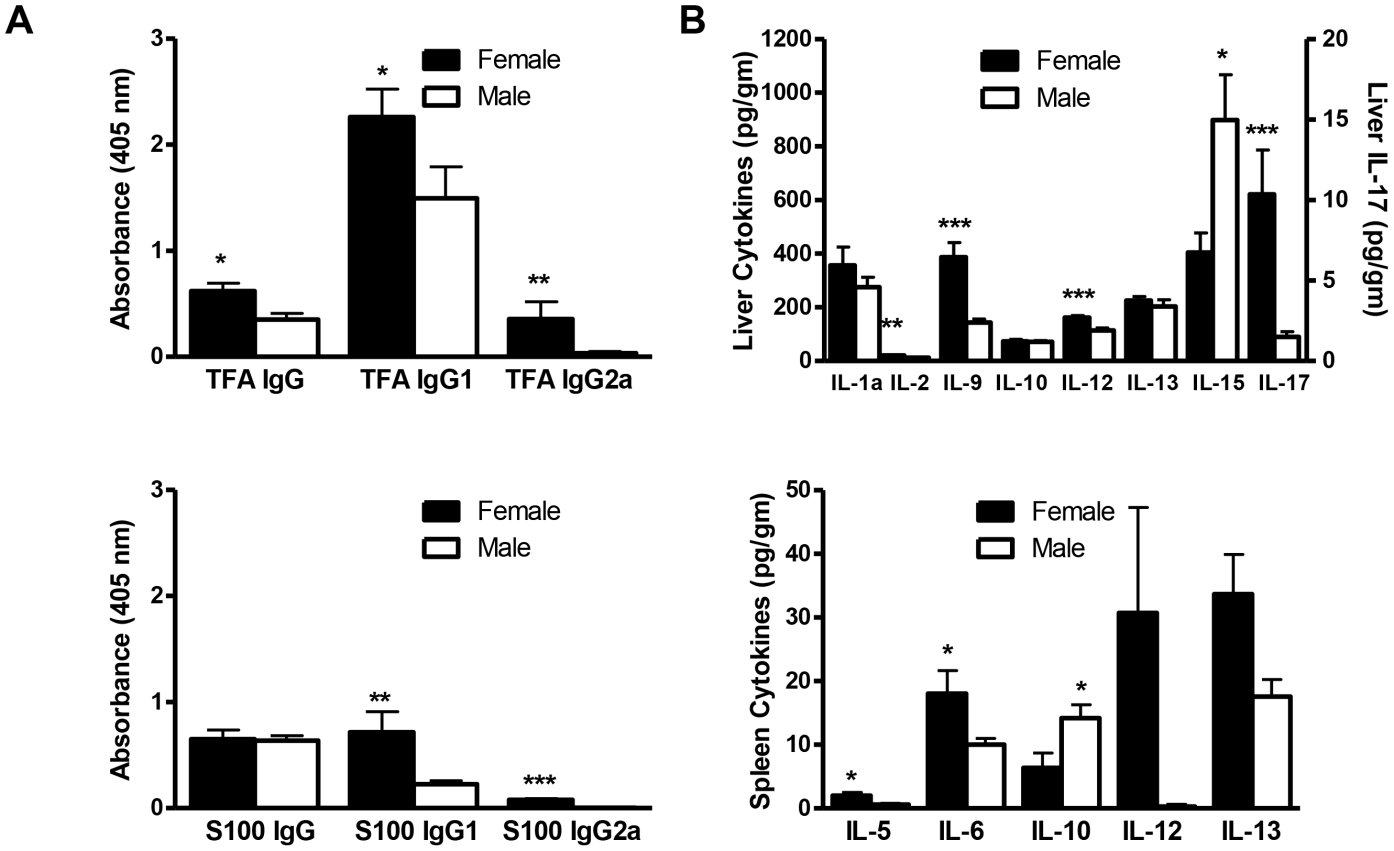
Experimental anesthetic DILI is associated with higher serum antibodies and proinflammatory cytokines in female BALB/c mice. (A) TFA IgG, IgG1, and IgG2a and S100 IgG1 and IgG2a antibodies were significantly higher in females than in males (values expressed as OD). (B) IL-2, IL-9, IL-12, and IL-17 (pg/g) were significantly higher in livers from females than in those from males. IL-5, IL-6, and IL-13 (pg/g) were significantly higher in spleens from females, but IL-10 was higher in spleens from males. mean ± SEM. *p<0.05, **p<0.01, ***p<0.001.

We first demonstrated sex bias in our experimental model of anesthetic immune-mediated hepatitis and then investigated its mechanisms during the priming and effector phases. After 3 weeks, hepatitis histology inflammation scores were higher in female (3.0±0.1, median ± SEM) than in male mice (1.0±0.2, p<0.01; Fig. 1A and 1B). At the same time point females had significantly more TCR+CD4+, TCR+CD8+, DX5+NK, and TCR+DX5+NKT lymphomyeloid leukocytes (Fig. 1C). TFA IgG, IgG1, and IgG2a antibodies and S100 IgG1 and IgG2a antibodies were also significantly elevated in females when compared to males (Fig. 2A). In our model, TFA IgG1 antibodies correlate with the severity of hepatitis [5]. Hence, finding significantly elevated TFA IgG1 antibodies in female mice strengthened results obtained by histology and flow cytometric analysis.

### IL-17 and other pro-inflammatory cytokines are increased in female BALB/c mice

Th17 cells have been linked to inflammatory and autoimmune diseases [24]–[26], and IL-17 correlates with severity of acute hepatic injury [27], [28]. Therefore, we examined whether the female bias in experimental anesthetic DILI is associated with increases in IL-17 and other pro-inflammatory cytokines.

After 3 weeks, IL-17, IL-2, IL-9, and IL-12 were significantly increased in the livers of female mice (Fig. 2B, upper panel). We have reported previously that this DILI model induces infiltration of neutrophils and mast cells into the liver [5]. It is possible that increased IL-17 could promote neutrophils while IL-9 sustains mast cell growth and survival [29]. We detected elevated IL-15 in the livers of male mice, a finding that could suggest protective roles for NK or NKT cells [30]; however, neither NK nor NKT cells were elevated in the male mice. We did not detect sex differences in hepatic TNF-α, IL-6, or IL-1β levels.

In contrast to the liver, the spleens of female mice exhibited increased IL-5, IL-6, and IL-13; IL-10 was significantly elevated in the spleens of male mice (Fig. 2B, lower panel). Elevated IL-5 and IL-13 support our prior findings that implicate IL-4-driven responses in this model [22]. Elevated IL-10 levels in males suggested that this cytokine directly or indirectly suppresses pro-inflammatory responses that induce hepatitis. However, male mice that received anti-IL-10 blocking antibodies did not exhibit enhanced hepatitis compared to mice that received isotype controls. To perform this experiment, male mice were immunized with TFA-S100 on days 0 and 7 as previously described in the Methods section and then treated with anti-IL-10 (250 ug) or isotype control on days 14, 16, 18 and 20 and then killed on day 21. We found that hepatitis as measured by inflammation score was not different between groups (0.7±0.1 (anti-IL-10) versus 0.8±0.5 (isotype), n = 4 BALB/c males/group, mean ± S.E.). This anti-IL-10 result suggested to us that anti-IL-10 did not have a direct effect on sex differences seen in our model and suggested to us an indirect effect of IL-10 that may help to explain worsening hepatitis in female mice.

### TFA-S100 immunizations impair splenic Treg expansion in female BALB/c mice

**Figure 3:**
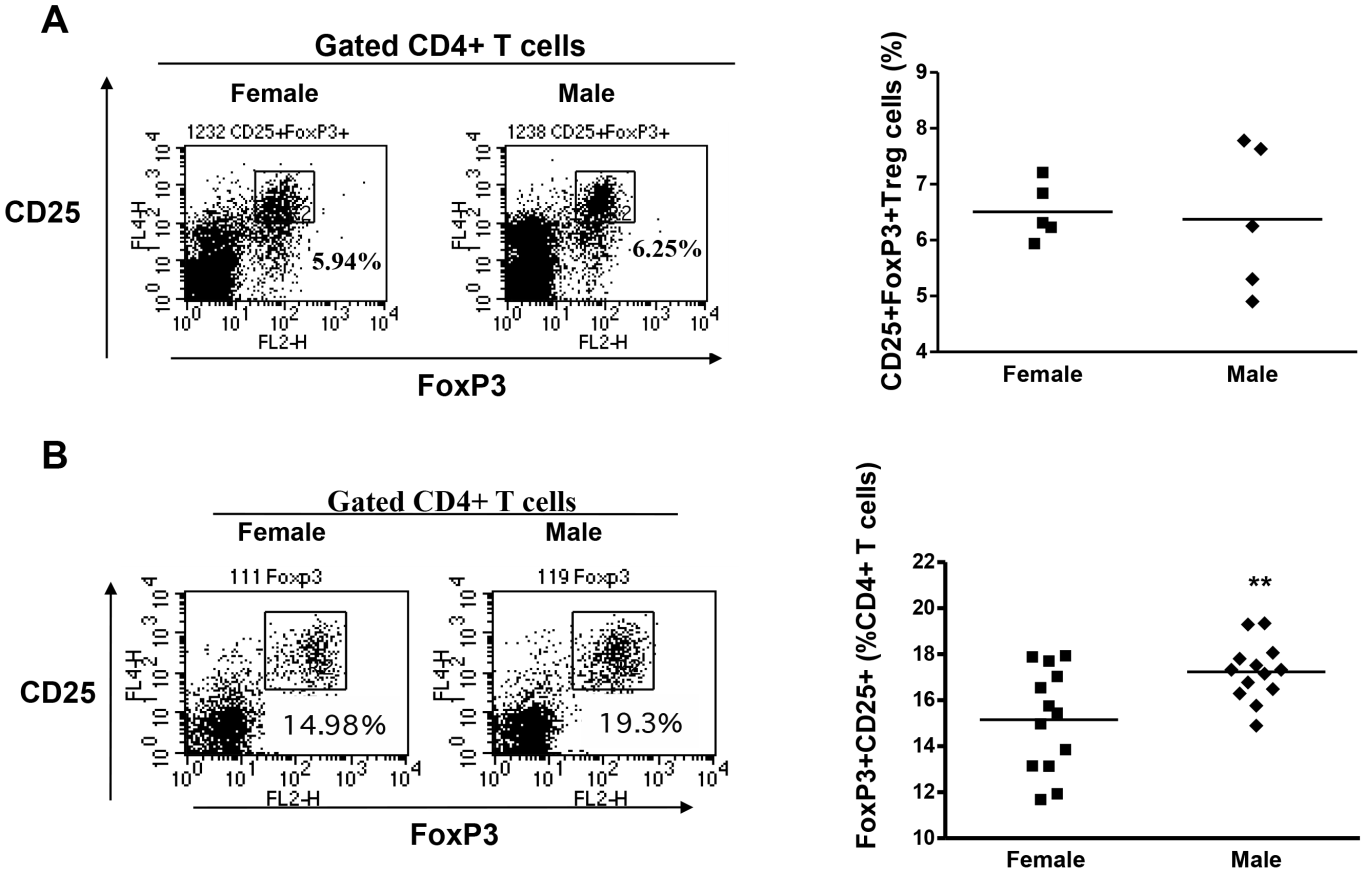
TFA-S100 immunizations impair splenic Treg expansion in female BALB/c mice. (A) Representative dot plot of gated CD4+ T-cells and scatter plot showing no sex differences in hepatic CD4+CD25+FoxP3+ cell (Treg) levels 3 weeks after TFA-S100 immunizations (n = 5/group). (B) Representative dot plot of gated CD4+ T-cells and scatter plot showing significantly elevated splenic Tregs in males 2 weeks after immunizations (Mann-Whitney *U*, n = 15/group *p<0.05, **p<0.01, ***p<0.001).

**Figure 4:**
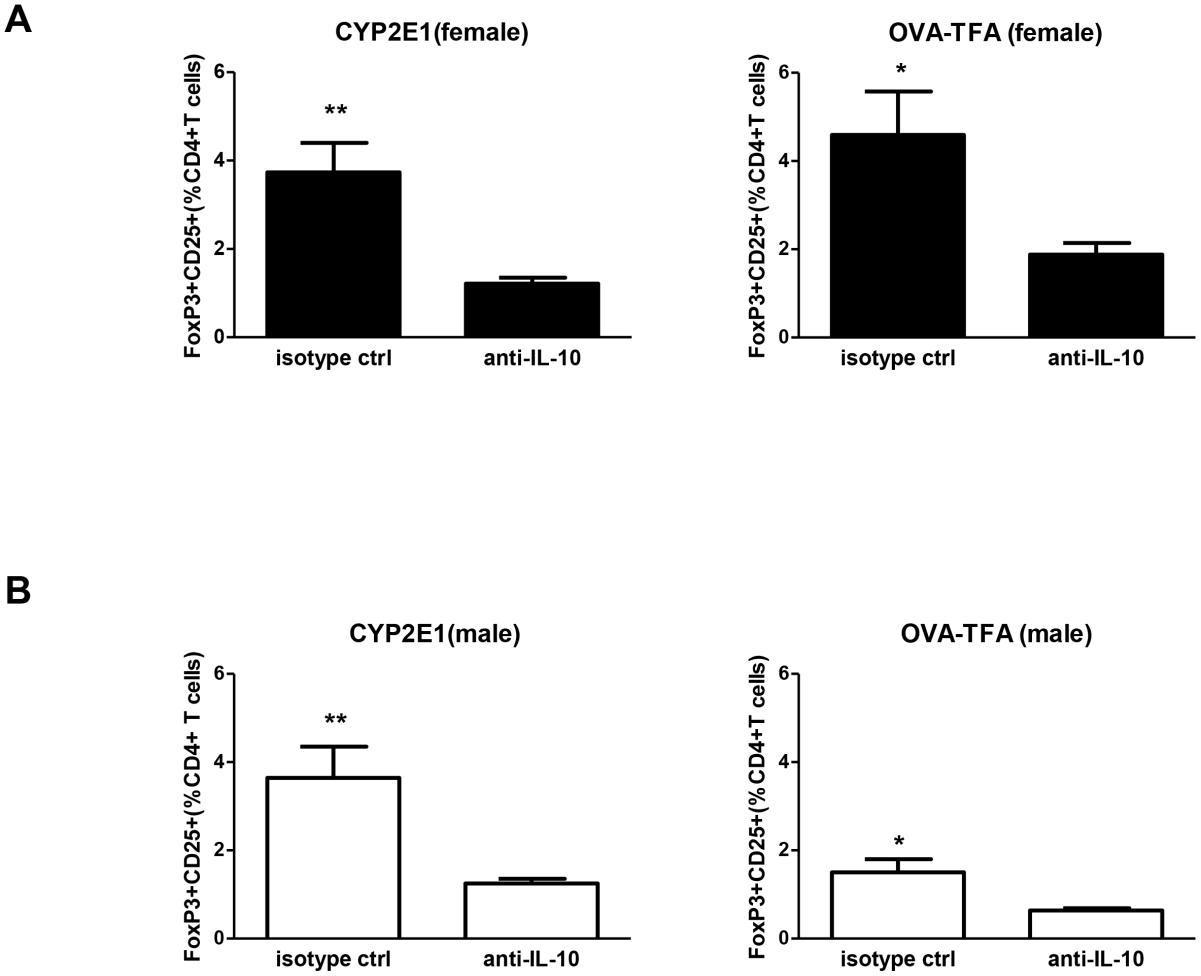
Anti-IL-10 antibody diminishes Tregs in TFA-S100 – immunized male and female splenocyte cultures in vitro. IL-10 blocking antibody (rat anti-mouse, 10 µg/mL), similarly diminished Tregs in female (A) and male (B) BALB/c mice isolated from TFA-S100- immunized mouse splenocytes challenged with CYP2E1 or the TFA (TFA-OVA) (Mann-Whitney *U*, *p<0.05, **p<0.01, ***p<0.001, experiments run in duplicate, n = 3 mice/group).

**Figure 5:**
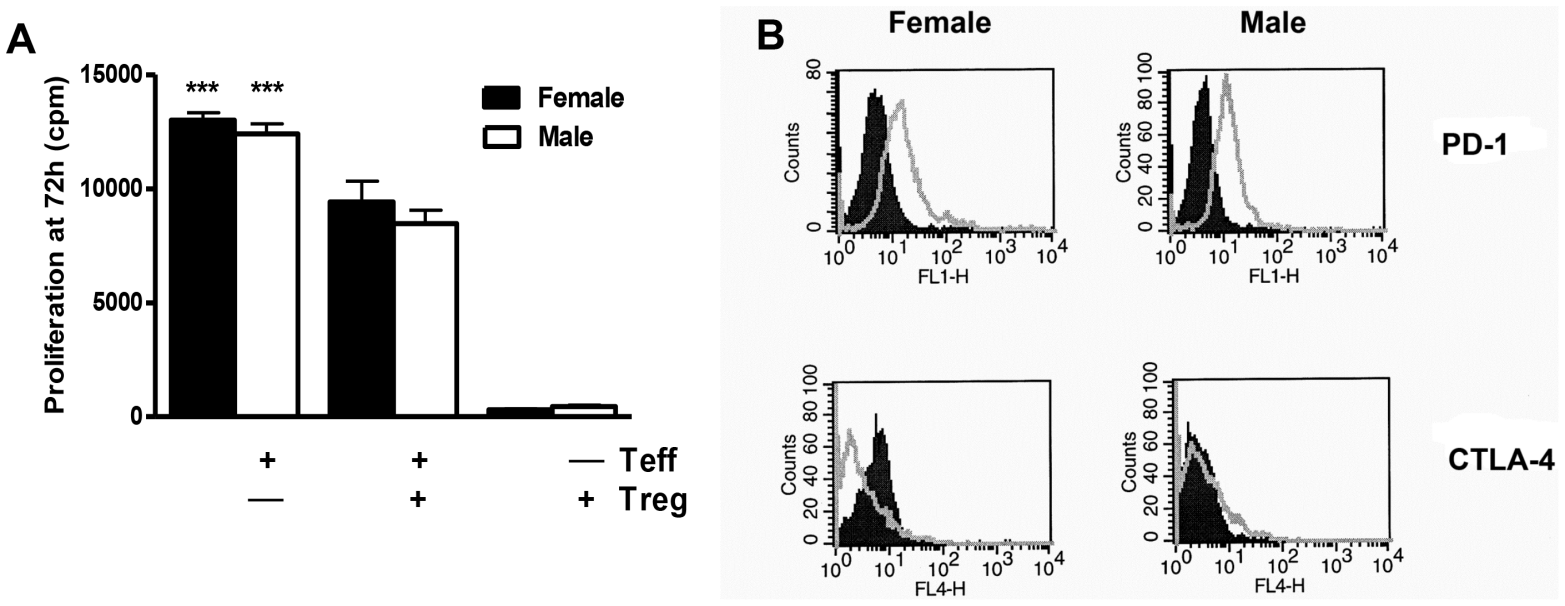
No sex bias in baseline Treg function in BALB/c mice. (A) There was no sex bias in proliferation of T-effector cells (n = 10/group) co-cultured with naïve Tregs (Treg∶T-effector ratio 1∶1) in the presence of anti-CD3 (5 µg/mL) and anti-CD28 (2.5 µg/mL). (B) Using FACS analysis, of Tregs co-cultured as described in A, intracellular expression of inhibitory molecules PD-1(CD279, cloneJ43) was similar in female and male mice, and CTLA-4 (CD152, cloneUC10-489) was not detected in either sex (Experiments run in duplicate, n = 5–6 mice/group naive = filled histogram, stimulated = open histogram *p<0.05, **p<0.01, ***p<0.001).

Interleukin-10 maintains and expands Tregs *in vitro* [31]. Therefore, we analyzed hepatic and splenic Tregs at different stages of anesthetic DILI. The number of hepatic Tregs was similar in the two sexes 3 weeks after the initial TFA-S100 immunization, during the peak of hepatitis (Fig. 3A). In contrast, the number of splenic Tregs was higher in male than in female mice after 2 weeks, during T-cell priming, (p<0.01, Fig. 3B). Interestingly, anti-IL-10 administration significantly reduced the number of Tregs in both sexes when splenocyte cultures from DILI mice were challenged with CYP2E1 (autoantigen) or TFA (hapten) (Fig. 4A and 4B). Next we compared the capacity of naïve BALB/c Tregs to inhibit proliferation of T-effector cells after inoculation with anti-CD3 and anti-CD28 *in vitro*. We found no sex bias in T-cell proliferation (Fig. 5A), indicating no sex bias in baseline Treg function. From these same cultures we also found that programmed death receptor-1, whose expression inhibits Treg proliferation [32], was only slightly lower in Tregs from female mice and that CTLA-4 was not detected in either sex (Fig. 5B) further suggesting that sex bias in our model may not be attributed to differences in Treg function [33] [34] [35]. These findings also suggest that quantitative differences in splenic Tregs during the priming phase of experimental DILI may contribute to the sex bias.

### IL-1β and IL-6 impair splenic Treg expansion in female BALB/c mice

By acting synergistically with other pro-inflammatory cytokines [36], IL-1β and IL-6 promote Th17 cells and inhibit Treg survival and Treg-mediated suppression of pro-inflammatory responses [37]. Because we have previously detected elevated splenic IL-6 levels in female mice (Fig. 2B, bottom panel), we compared IL-1β, IL-6, and Tregs in splenocyte cultures from naïve and TFA-S100-immunized BALB/c mice.

**Figure 6:**
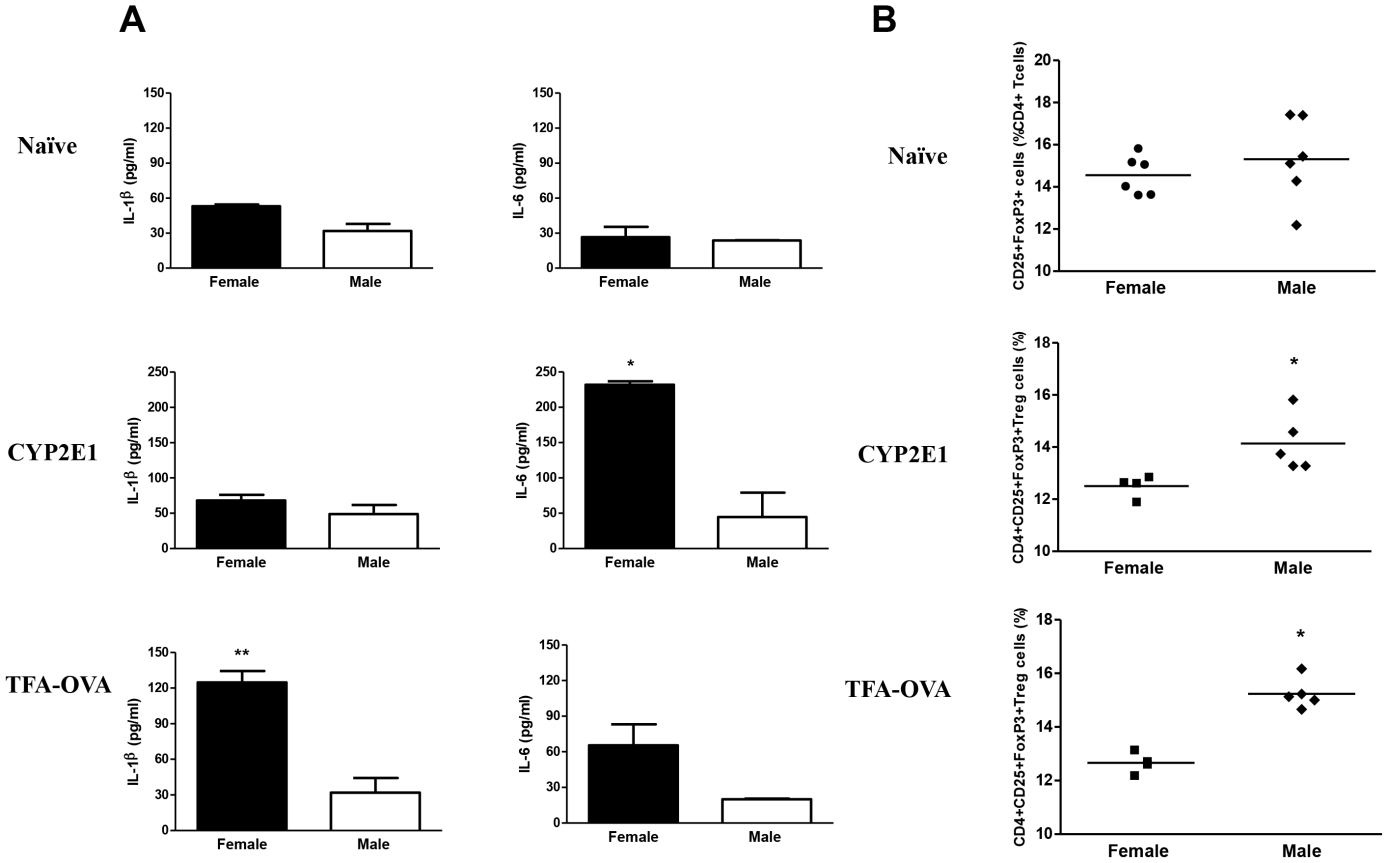
IL-1β and IL-6 impair splenic Treg expansion in female BALB/c mice. (A) Naïve mice exhibited no sex bias in IL-6 or IL-1β level or in number of Tregs; however, IL-6 and IL-1β were significantly elevated in splenocytes isolated from female mice 2 weeks after immunization and stimulated with CYP2E1 or TFA *in vitro* (10 µg/mL; Data are shown as mean ± SEM. Experiments run in duplicate n = 5/group). (B) The number of Tregs was significantly reduced in cultures from females when compared to males. (Experiments run in duplicate, n = 3 mice/group, Mann-Whitney *U*, * = p<0.05, ** = p<0.01, *** = p<0.001).

**Figure 7:**
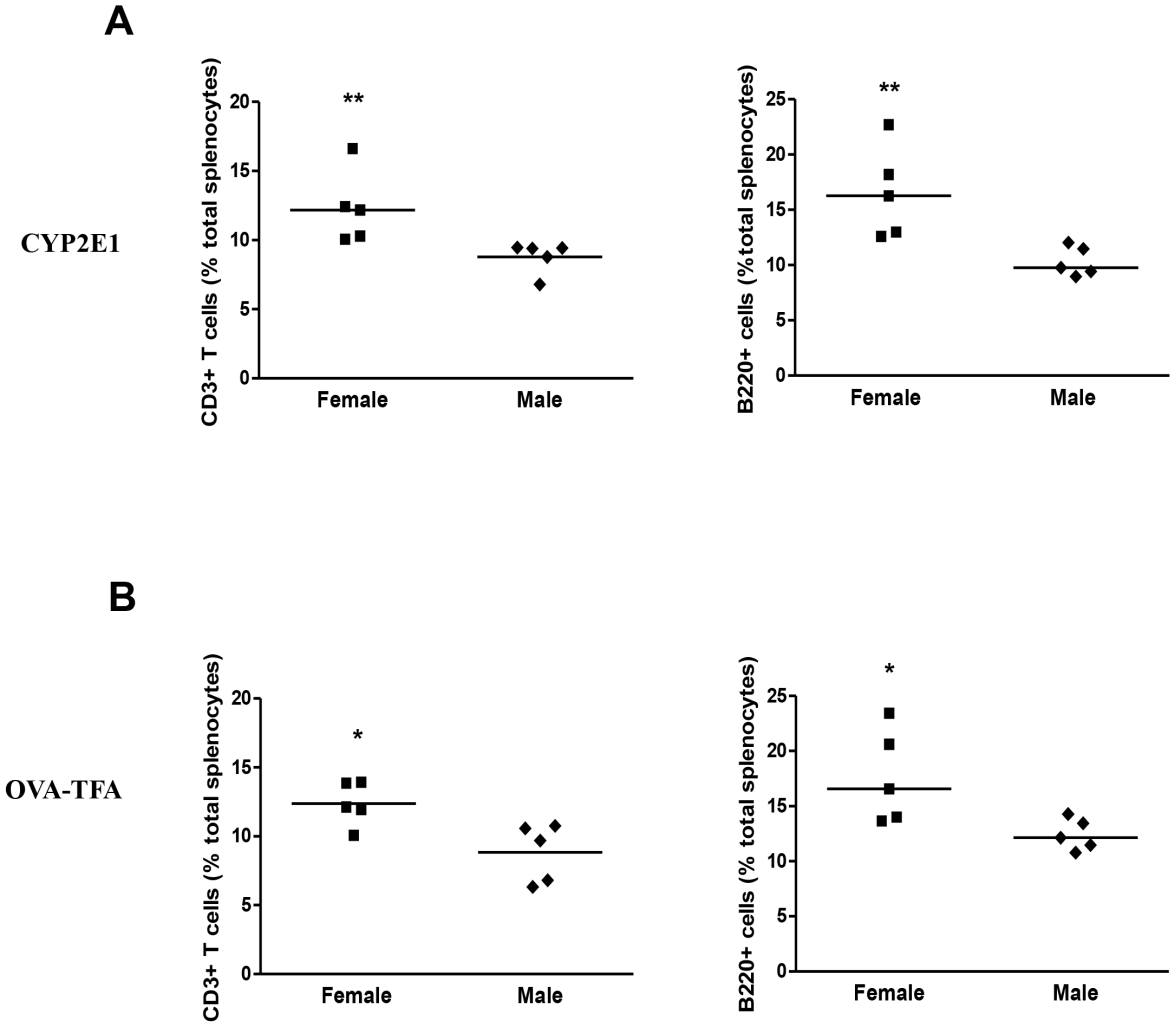
Increased T and B cells are associated with impaired splenic Treg expansion in female BALB/c mice following TFA-S100 immunizations. Splenocytes were isolated from female mice 2 weeks after TFA-S100 immunizations, stimulated with CYP2E1 (A) or TFA (B) *in vitro* (10 µg/mL) and analyzed for Tregs as described in Figure 6. CD3+CD25 T cells and B220+ cells were significantly elevated in the same cultures from females when compared to males. (Experiments run in duplicate, n = 3 mice/group, Mann-Whitney *U*, * = p<0.05, ** = p<0.01, *** = p<0.001).

Naïve male and female mice produced equal amounts of IL-1β, IL-6, and Tregs (Fig. 6A and 6B). However, during T-cell priming, splenocytes from females produced higher IL-6 levels after challenge with CYP2E1 (p<0.05) and higher IL-1β levels after TFA challenge than did splenocytes from males (p<0.01, Fig. 6A). Correspondingly, we detected lower Treg levels in these same cultures (p<0.05, Fig. 6B). The numbers of T- and B-cells were significantly increased in these cultures (Fig. 7A and 7B), indicating that the decrease in number of Tregs in female splenocyte cultures was not the result of lower total cell numbers. These findings suggest that splenic production of cytokine IL-6 triggered by the CYP2E1 autoantigen or IL-1β triggered by the TFA hapten may inhibit Treg expansion. Additionally, IL-1β and IL-6 responses demonstrated in female mice might be regulated by estrogen [21], [22].

**Figure 8:**
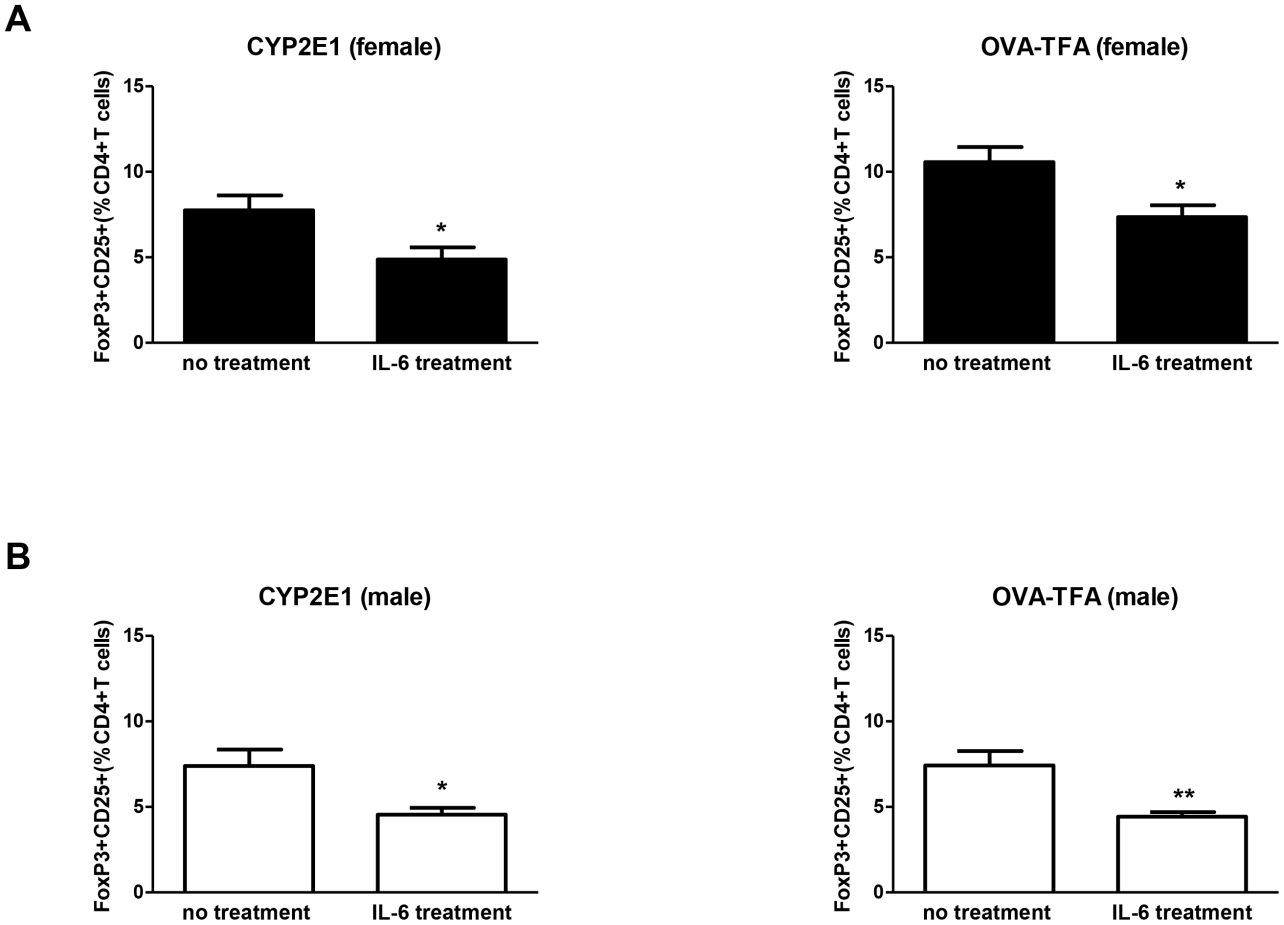
IL-6 supplementation diminishes Tregs in TFA-S100 – immunized male and female splenocyte cultures in vitro. IL-6 supplementation (25 ng/mL) of *in vitro* splenocyte cultures from female (A) and male (B) TFA-S100-immunized BALB/c mice diminished Tregs in both groups. (Experiments run in duplicate, n = 3 mice/group, Mann-Whitney *U*, * = p<0.05, ** = p<0.01, *** = p<0.001).

To first investigate autoimmune pathways in experimental DILI, we confirmed no female bias in Treg responses to IL-6 by reducing Tregs in CYP2E1- and TFA-stimulated splenocytes cultured from both male and female mice with experimental DILI (Fig. 8A and 8B). These studies support our contention that sex bias in experimental DILI is promoted by lower Treg levels in females, not by differences in Treg function.

**Figure 9:**
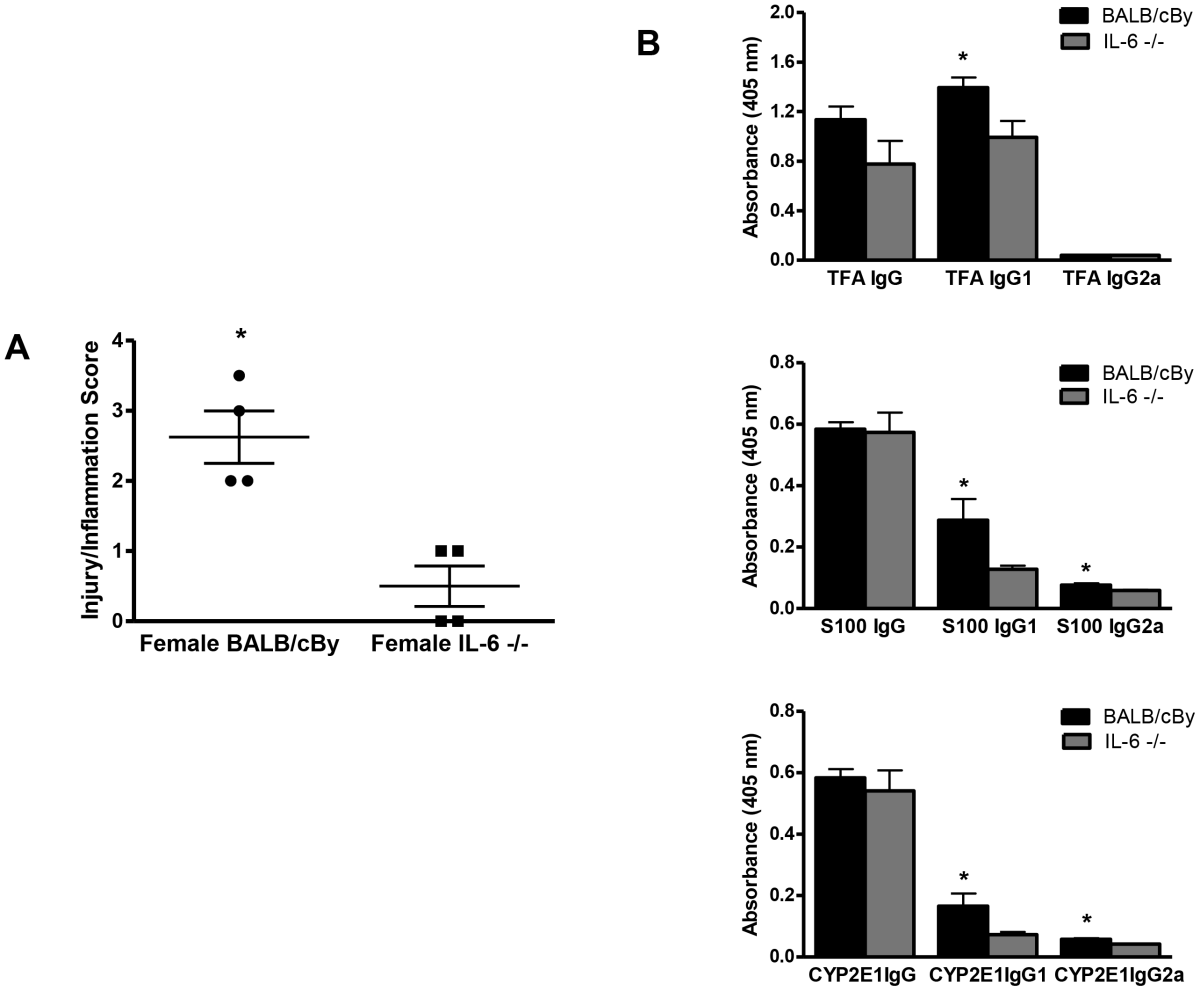
Anesthetic DILI is diminished in IL-6 deficient (−/−) mice. IL-6 −/− mice were immunized with TFA-S100 as described in the Methods section. (A) Twenty one days later, IL-6−/− mice developed significantly less hepatic injury (0.5±0.3, mean ± SEM) than did BALB/cBy mice (2.5±0.4). (B) IL-6−/− mice also produced lower TFA, S100, and CYP2E1 IgG1 and S100 and CYP2E1 IgG2a antibody levels than did BALB/cBy controls (n = 4/group, Mann-Whitney *U*, * = p<0.05).

**Figure 10:**
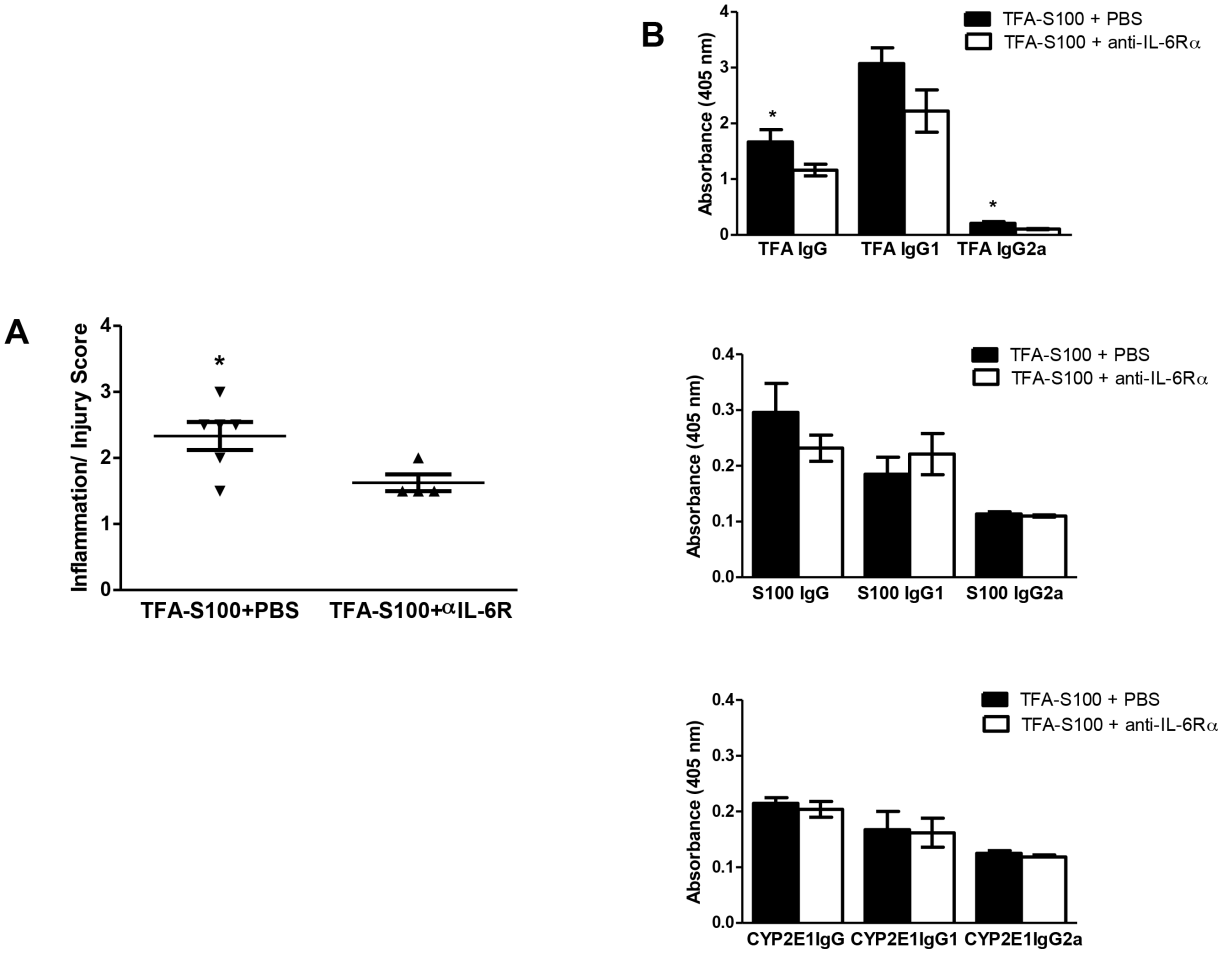
IL-6Rα antibodies diminish anesthetic DILI in female BALB/c mice. Female BALB/c mice were immunized with TFA-S100 as described in the Methods section. (A) IL-6Rα antibodies administered days 0 and 7 diminished hepatitis as demonstrated by decreased inflammation/injury scores (1.6±0.1, mean ± SE) when compared to TFA-S100 – immunized, female , BALB/c mice not treated with IL-6Rα but administered phosphate buffered saline at the same time points (2.3±0.2, mean ± SE). (B) Anti-TFA IgG and IgG2a subclass antibody levels measured in mouse sera were also decreased (p<0.05) when compared to mice immunized with TFA-S100 without blocking antibodies. (B) S100 and (C) CYP2E1 autoantibody levels were not significantly different when comparing sera of mice immunized with TFA-S100 ± anti-IL-6Rα. (Mann – Whitney *U*, n =  5/group, *p<0.05).

To uncover an additional role for IL-6, we immunized female IL-6−/− mice that are on a BALB/cBy background [5]. Hepatitis histologic scores (Fig. 9A, p<0.05); TFA, S100, and CYP2E1 IgG1 antibody levels; and S100 and CYP2E1 IgG2a antibody levels (Fig. 9B) were lower in IL-6−/− mice than in BALB/cBy mice. However, levels of splenic and hepatic cytokines were not statistically different between the two groups. These data suggest that splenic IL-6 promotes hepatitis and antibody production in immune-mediated DILI; however whether or not estrogen has a role in modulating IL-6 is not clear. Moreover, IL-6−/− mice have hematopoietic deficiencies that may include other cell lines such as B cells that may also be critical to developing hepatitis following TFA-S100 immunizations. For this reason we utilized IL-6Rα antibodies in order to more effectively diminish the actions of IL-6 in BALB/c mice [38]. We found that treatments with IL-6Rα antibodies during the induction of our model significantly diminished hepatitis (p<0.05) as demonstrated by decreased inflammation/injury scores (1.6±0.1, mean ± S.E.) when compared to TFA-S100 – immunized BALB/c mice not treated with this antibody but administered phosphate buffered saline at the same time points (2.3±0.2, mean ± S.E., Fig 10A). Anti-TFA IgG and IgG2a subclass antibodies measured in mouse sera were also decreased (p<0.05) when compared to mice immunized with TFA-S100 without blocking antibodies (Fig. 10B). However, the effect was not as striking as that seen with IL-6 deficient mice (Fig. 9). This finding suggested to us that IL-6 has a role in the severity of hepatitis and anti-TFA (hapten) antibodies. However, generation of S100 and CYP2E1 autoantibodies were not directly affected by IL-6 and estrogen may have a greater role by itself or in concert with other factors.

### Castration of BALB/c mice modulates experimental immune-mediated DILI

**Figure 11:**
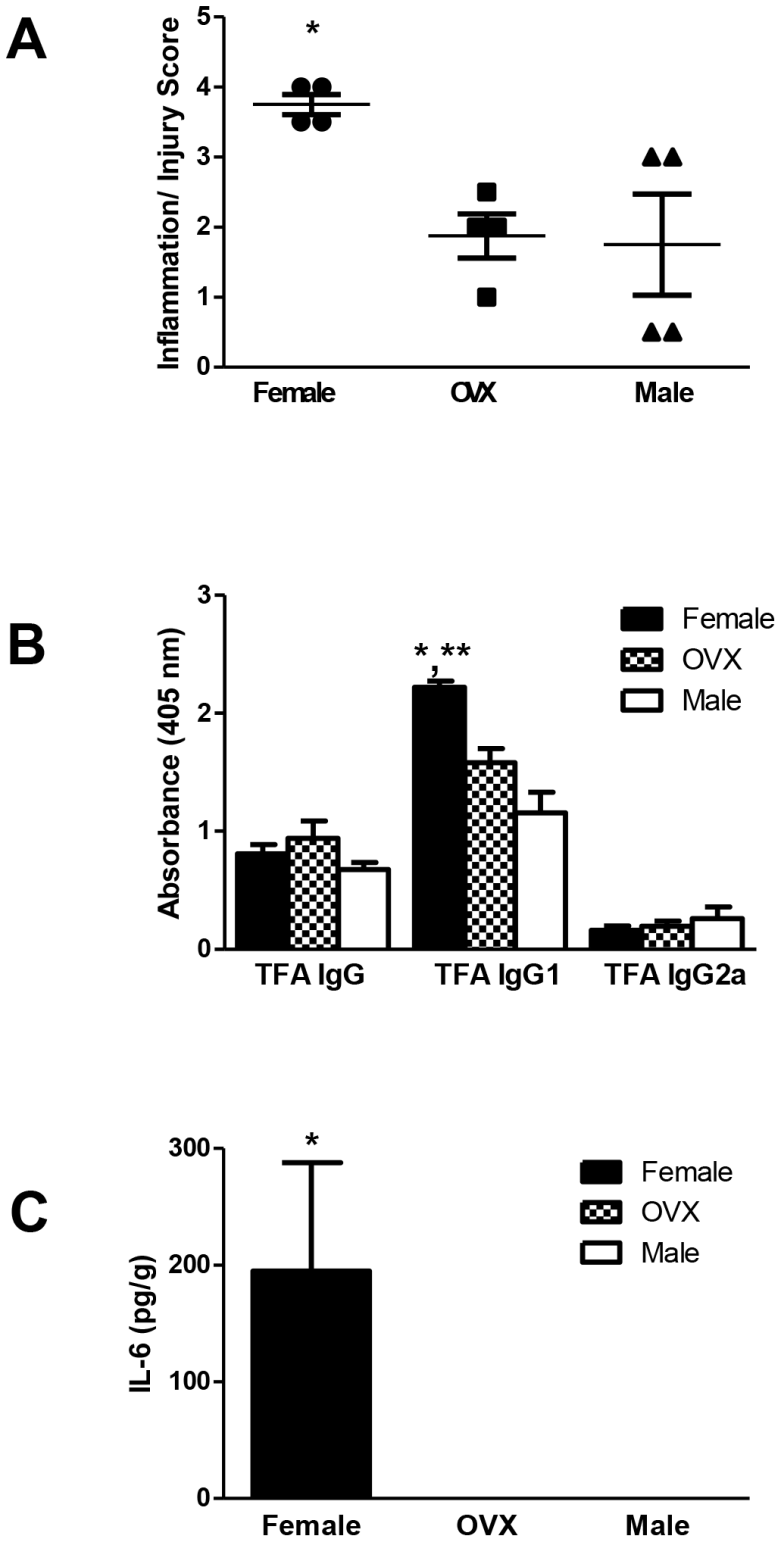
Ovariectomy reduces anesthetic DILI in female BALB/c mice. Intact BALB/c females and males as well as females who had undergone ovariectomy were immunized with TFA-S100 as described in the Methods. By 3 weeks, the intact females had developed significantly more severe hepatitis (A, *p<0.05) and exhibited significantly higher levels of TFA IgG1 antibody levels (B) compared to ovariectomized (OVX, *p<0.05) or male mice (**p<0.01, n = 4 mice/group). (C) Splenic IL-6 levels were significantly higher in intact female mice than in OVX or male mice at 2 weeks after immunizations. (Mann-Whitney *U*, * = p<0.05, ** = p<0.01).

To determine if estrogens increase the severity of experimental, immune-mediated DILI, we first immunized BALB/c ovariectomized mice. Hepatitis histology scores were similar in ovariectomized and non-ovariectomized mice at 2 weeks; however, by 3 weeks, scores were higher in intact BALB/c females than in ovariectomized or male mice (Fig. 11A, p<0.05). TFA IgG1 antibodies also were significantly elevated in BALB/c females (Fig. 11B), confirming that hepatitis was more severe in this group [5]. Notably, at 2 weeks, splenic IL-6 was the only cytokine significantly higher in BALB/c females than in the other groups (Fig. 11C), but by 3 weeks, splenic and hepatic cytokines were not significantly different among the three groups.

**Figure 12:**
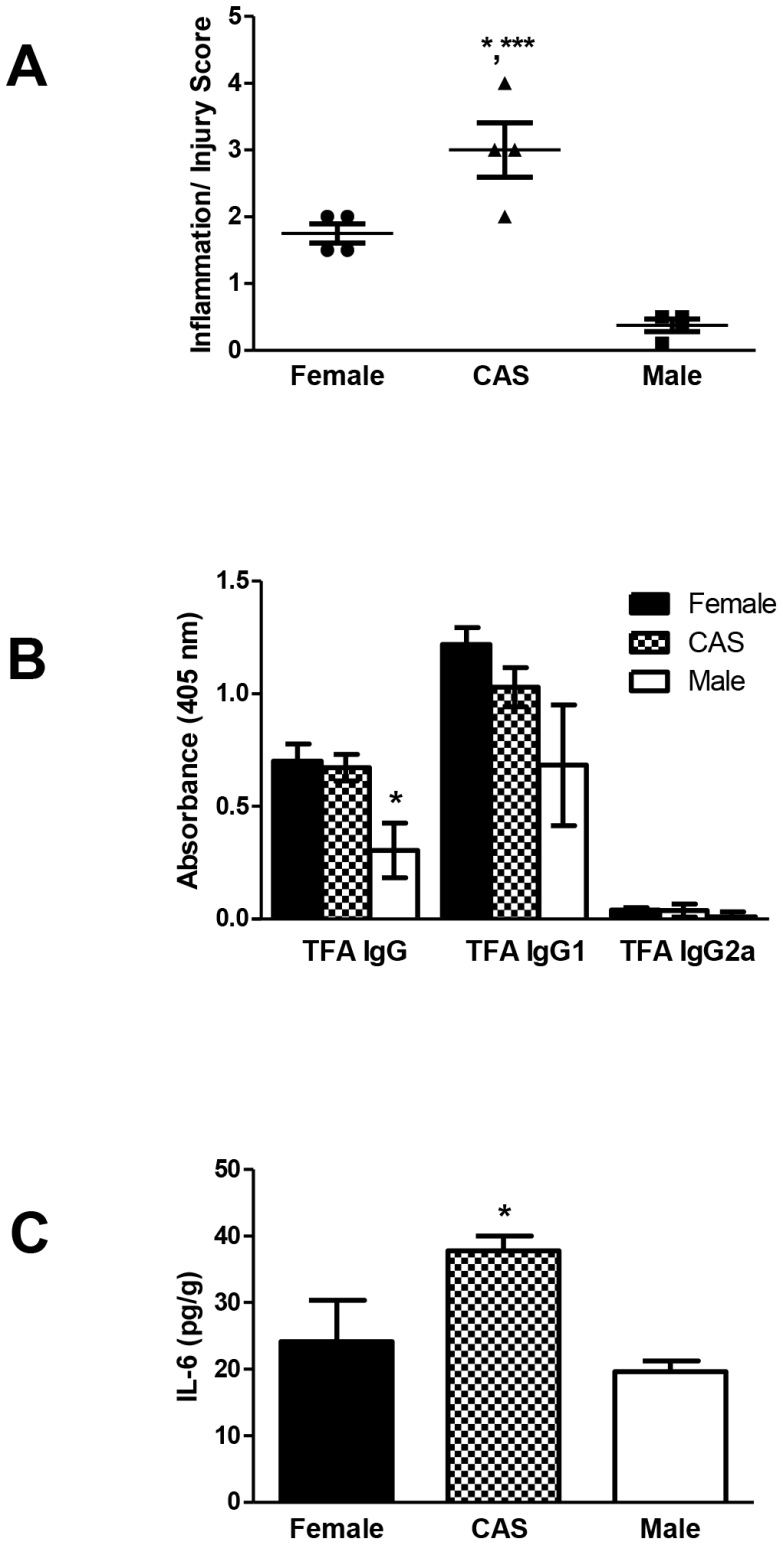
Elevated IL-6 levels are associated with more severe anesthetic DILI in castrated male BALB/c mice. Intact BALB/c females and males, as well as males who that had undergone castration, were immunized with TFA-S100 as described in the Methods section (A) By 3 weeks, castrated males (CAS) had developed significantly more severe hepatitis than females (*p<0.05) and intact males (***p<0.001). (B) Intact males exhibited significantly lower levels of TFA IgG antibodies when compared to females or intact male mice (*p<0.05, n = 4 mice/group). (C) Splenic IL-6 levels were significantly higher in CAS than in intact male mice. (Mann-Whitney *U*, * = p<0.05, *** = p<0.001).

To determine if castration of BALB/c males would also modulate hepatitis severity and antibody production, we immunized castrated BALB/c males as described, using BALB/c females and intact males as controls. Evaluation of the mice 3 weeks after immunization revealed that the severity of experimental DILI was markedly greater in castrated males than in non-castrated males (p<0.001) or BALB/c females (p<0.05, Fig. 12A). TFA IgG antibodies were lower in sera from intact males when compared to all other groups (p<0.05, 12B); however, significant differences in CYP2E1 levels were not found (not shown). Additionally, splenic IL-6 levels were elevated in castrated males when compared to intact males (p<0.05, Fig 12C).

**Figure 13:**
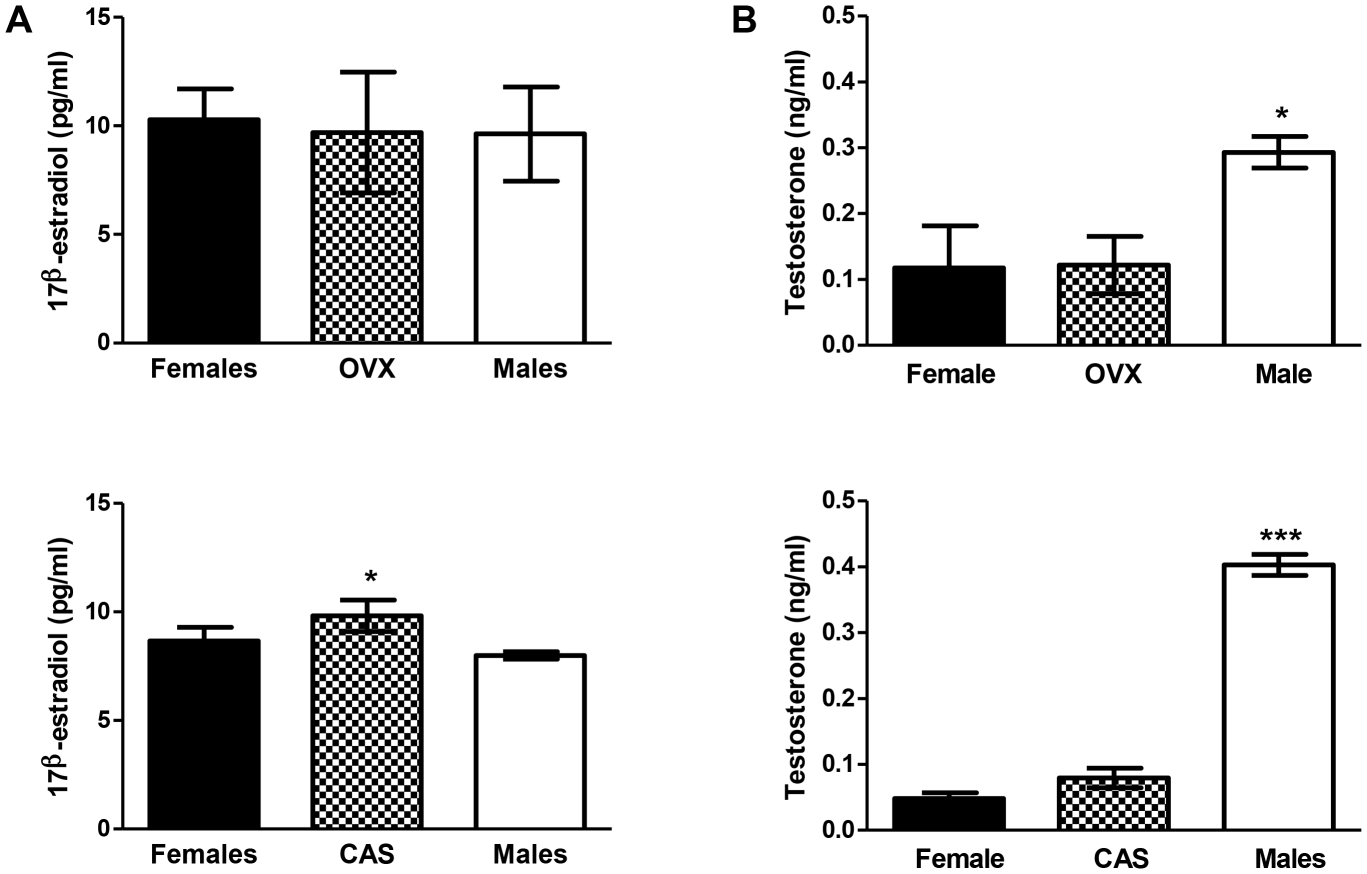
Elevated 17β-estratiol levels are associated with more severe anesthetic DILI in castrated male BALB/c mice. (A) Serum 17β-estradiol levels (pg/mL) were similar when comparing intact females, OVX and male mice (top panel); however serum 17β-estradiol levels were higher in CAS when compared to intact male mice (bottom panel, *p<0.05). (B) Serum testosterone levels (ng/mL) were higher in male mice when compared to intact female or OVX (top, ***p<0.001) and when compared to female or CAS (bottom, ***p<0.001), (Mann-Whitney *U*, * = p<0.05, *** = p<0.001).

To determine roles for circulating 17β-estradiol (E2) or testosterone, we measured these parameters in sera from all mice treated in the ovariectomy and castration experiments. When assessing roles for ovariectomy, we found that E2 was similar in all groups; however when assessing roles for castration of male mice in immune-mediated DILI, we found that castrated males had significantly higher E2 levels than did intact males (Fig. 13A, bottom panel). Although we expected testosterone levels to be significantly higher in males than in females, we also found that testosterone levels were higher in males than in ovariectomized females (p<0.05, Fig. 13B, top panel) and castrated males (p<0.01, Fig. 13B, bottom panel). These data suggest that higher estrogen levels may have increased severity of DILI in castrated males and indicate that testosterone may have a protective role.

**Figure 14:**
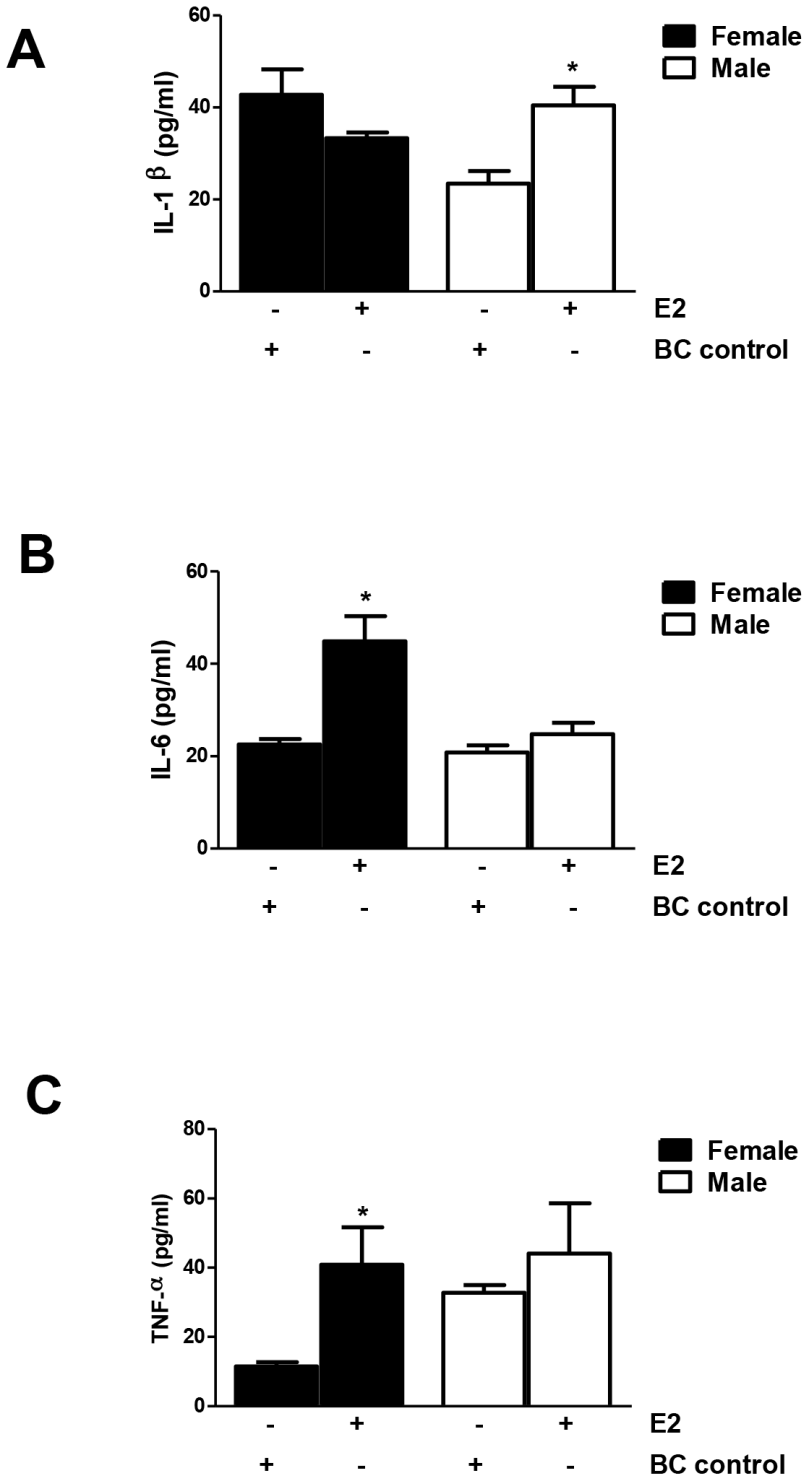
17β-estradiol (E2) induces pro-inflammatory cytokines in naïve splenocytes from BALB/c mice. 17β-estradiol (E2, 50 nM) increased IL-1β production in splenocytes cultured from naïve male BALB/c mice when compared to splenocytes cultured from naïve male BALB/c mice treated with β-cyclodextrin (BC, 5 mM) as a control (A, *p<0.05). E2 significantly increased IL-6 (B) and TNF-α (C) production in splenocytes cultured from naïve females when compared to splenocytes from naïve females treated with BC (*p<0.05). (Experiments run in duplicate, n = 5/group, Mann-Whitney *U*, * = p<0.05).

Because serum estrogen levels and *in vivo* castration experiments did not completely clarify the role of estrogen in DILI, we directly investigated the effect of estrogen on IL-6 by analyzing naïve BALB/c splenocytes cultured with E2 or β-cyclodextrin (control) for 24 h *in vitro*. We similarly measured the effect of E2 on IL-1β and TNF-α because these cytokines also have an immunomodulatory effect on Tregs. E2 induced IL-6 and TNF-α secretion by splenocytes from female but not from male mice, whereas IL-1β was induced in splenocytes from males (p<0.05, Fig. 14A–14C). Taken together, these data strongly suggest that E2 may increase the severity of experimental DILI by upregulating IL-6 and possibly TNF-α; however no sex bias in TNF-α levels was demonstrated in experimental DILI (Fig. 2B). Along the same lines, we could not demonstrate sex bias in IL-1β using our in vivo model (Fig 2B).

### Tregs modulate experimental anesthetic DILI

To determine whether Treg supplementation ameliorates disease, we immunized female BALB/c mice with TFA-S100 on days 0 and 7. At 2 weeks (day 14), mice received either intravenous PBS or 1×10^6^ freshly isolated IL-7-low, CD4+CD25+ cells from congenic naïve female mice. One week later (day 21), we evaluated hepatitis, cytokines, and antibodies.

**Figure 15:**
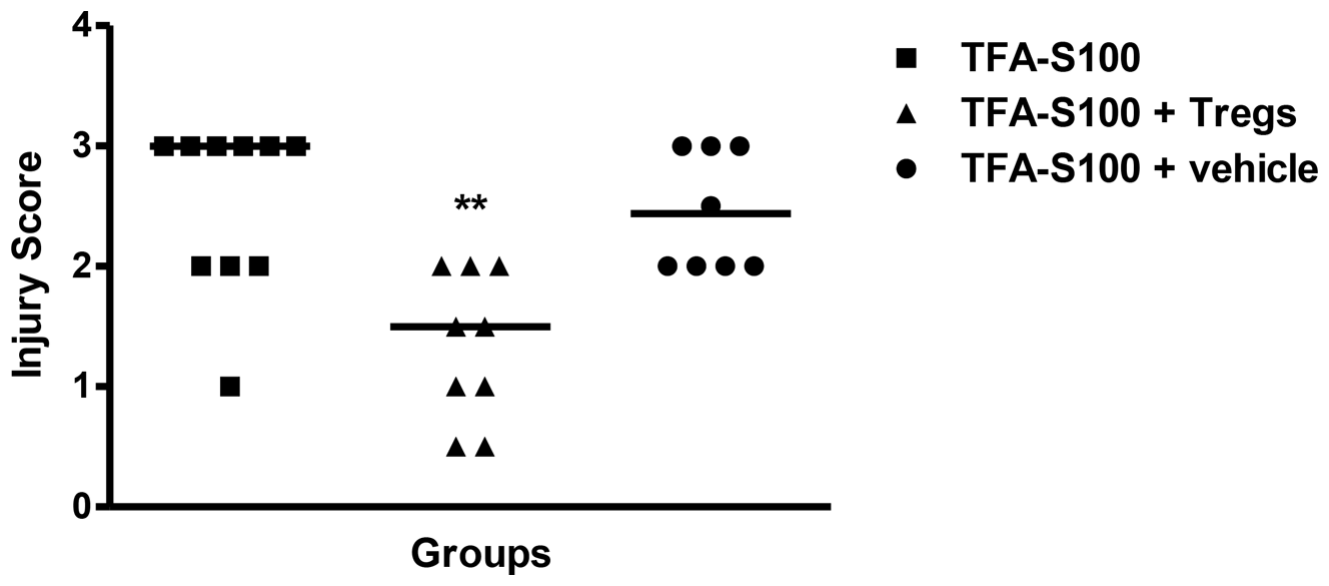
Tregs reduce experimental anesthetic DILI in female BALB/c mice. Two weeks after the TFA-S100 immunizations, mice were given intravenous injections of 1×10^6^ freshly isolated Tregs in PBS or PBS alone (control); the mice were killed 7 days later. The degree of hepatitis was significantly reduced in mice administered Tregs compared to those administered vehicle. Data are shown as mean ± SEM., n = 8–10 mice/group, Mann-Whitney *U*, *p<0.05, **p<0.01, ***p<0.001.

**Figure 16:**
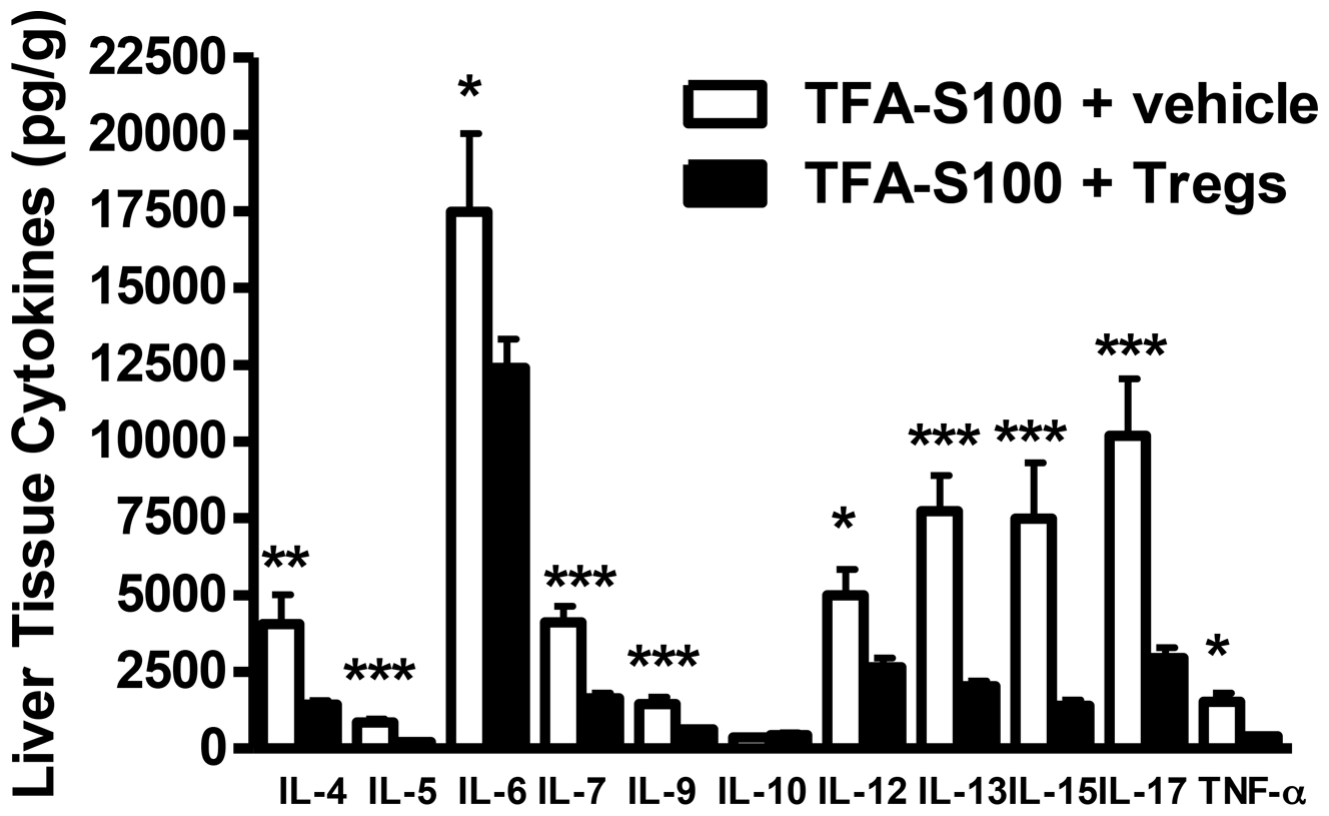
Tregs reduce hepatic pro-inflammatory cytokine levels following the induction of experimental anesthetic DILI in female BALB/c mice. Liver supernatants were analyzed from BALB/c females immunized with TFA-S100 ± Tregs. IL-4, IL-5, IL-6, IL-7, IL-9, IL-12, IL-13, IL-15, IL-17, and TNF-α (pg/g) were significantly lower in livers of Treg-treated mice. Data are shown as mean ± SEM., n = 8–10 mice/group Mann-Whitney *U*, *p<0.05, **p<0.01, ***p<0.001.

**Figure 17:**
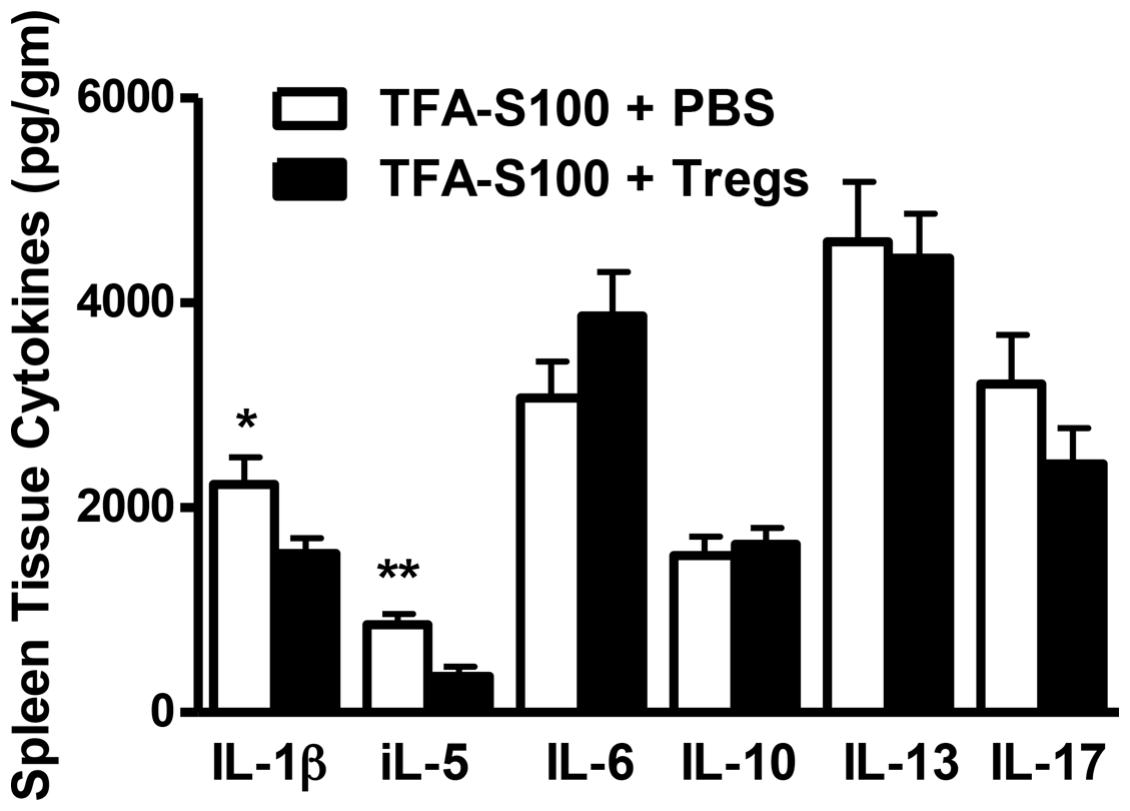
Tregs reduce splenic IL-1β and IL-5 levels following the induction of experimental anesthetic DILI in female BALB/c mice. Spleen supernatants were analyzed from BALB/c females immunized with TFA-S100 ± Tregs. IL-1β and IL-5 were significantly lower in spleens of Treg-treated mice when compared to levels in vehicle-treated mice. Data are shown as mean ± SEM., n = 8–10 mice/group, Mann-Whitney *U*, *p<0.05, **p<0.01, ***p<0.001.

**Figure 18:**
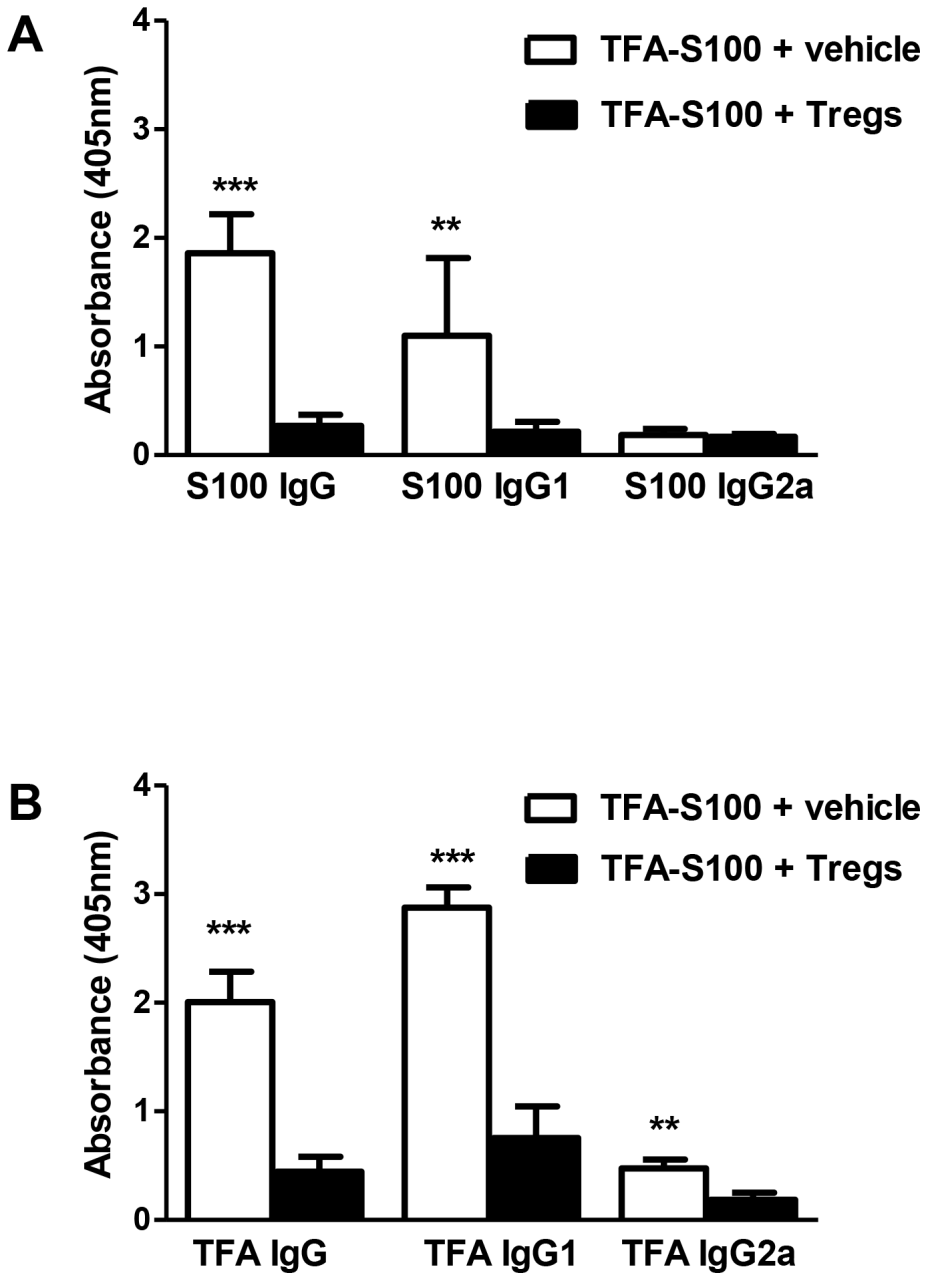
Tregs reduce serum S100 and TFA antibody levels following the induction of experimental anesthetic DILI in female BALB/c mice. Spleen supernatants were analyzed from BALB/c females immunized with TFA-S100 ± Tregs. S100 IgG and IgG1 (A), TFA IgG and IgG1, and TFA IgG2a. (B) Antibodies were lower in the sera from Treg-treated group than in controls. Data are shown as mean ± SEM., n = 8–10/group, Mann-Whitney *U*, *p<0.05, **p<0.01, ***p<0.001.

Adoptive transfer of Tregs significantly reduced the degree of hepatitis to levels seen in male mice (Fig. 15). Hepatic IL-4, IL-5, IL-6, IL-7, IL-9, IL-12, IL-13, IL-15, IL-17 and TNF-α (Fig. 16) and splenic IL-1β and IL-5 (Fig. 17) were significantly decreased in Treg-treated mice. Antibodies, including TFA IgG1, were also significantly decreased in these mice (Fig. 18A and 18B). Thus, our data strongly suggest that Treg modulation of inflammatory signaling during the priming phase of experimental anesthetic DILI is a critical step in pathogenesis of sex bias.

## Discussion

In this study we found, that similar to patients with anesthetic DILI, female BALB/c mice develop more severe experimental, drug-hapten, immune-mediated DILI than do males. We advance understanding of the immunological basis of this disease by demonstrating that estrogen induces IL-6 in female BALB/c mice and that IL-6-promoted impairment of splenic Treg expansion may determine the severity of experimental DILI. We also show that adoptive transfer of additional Tregs into female BALB/c mice reduces the degree of hepatitis and the production of pro-inflammatory cytokines and TFA IgG1 antibodies. To our knowledge, this is the first demonstration of shared roles for IL-6 and Tregs in a mouse model of immune-mediated DILI induced by drug haptens. We also provide a mechanism that explains, in part, sex bias that occurs in this disease.

Sex bias has been demonstrated previously in concanavalin A immune-mediated hepatitis [39], in which female BALB/c mice develop more severe hepatitis and exhibit higher levels of plasma TNF-α, IFN-γ, and IL-4 than do males. We have similarly demonstrated increased severity of experimental drug-hapten hepatitis in female BALB/c mice. However, our data show higher splenic IL-6 in females and higher IL-10 in males. Thus, our studies confirm, as did the landmark study by Takamoto et al. [39], the importance of sex in the pathogenesis of immune-mediated hepatitis, but our cytokine data suggest that alternative mechanisms regulate sex bias in immune-mediated, drug-hapten–induced DILI.

As in concanavalin A hepatitis, CD4+ T-cells regulate immune responses in anesthetic DILI [22]; however, in anesthetic and other drug-induced DILI, hepatitis is predominantly neutrophilic. In a previously published review, Mills [40] described how IL-17–secreting T-cells promote neutrophilic inflammation by triggering IL-1β and IL-6. We also have found increased IL-17, IL-1β, and IL-6 in female BALB/c mice and previously suggested key roles for IL-9, a promoter of mast cells, and IL-13, the effector cytokine for IL-4 [21]. Taken together with prior reports, our findings suggest that alternative cytokine pathways triggered by drug haptens induce predominantly granulocytic hepatitis.

Pro-inflammatory cytokines determine whether T-cell precursors become regulatory or pro-inflammatory Th17 cells [41], [42]. Thus, increased splenic IL-6, IL-1β, or even TNF-α could explain why the number of Tregs is lower in females than in males, even without confirming Th17 cells in these mice. Pro-inflammatory cytokines IL-1β, IL-6, and TNF-α synergistically suppress the proportion and function of Tregs [36]. Although we did not find sex bias in TNF-α expression, our studies support this notion by showing that naive females and males produce almost equal amounts of splenic IL-6 and IL-1β but that TFA-S100 immunizations induce females to produce significantly more than males (Fig. 6A and 6B). Hence, we would expect no sex differences in Tregs at baseline, but after TFA-S100 immunizations, Tregs would be expected to be higher in males (Fig. 3B). Previous reports have shown that splenic dendritic cells also regulate Treg suppressive activity [37]. In this study we did not isolate the source of IL-6 or IL-1β, but we are currently pursuing these investigations.

In our study, castration exacerbated the severity of hepatitis and increased TFA IgG antibody levels of male mice after TFA-S100 immunization. In addition, castrated males had significantly higher E2 levels than did intact males. Our data highlight potential roles for E2-induced IL-6 and lowered Treg levels in worsening experimental anesthetic DILI in female mice. By demonstrating no difference in hepatitis priming between intact females, ovariectomized females, and male mice at 2 weeks, we show that E2 may not cause sex bias directly but may enhance immune responses once antigen recognition occurs. Undetectable levels of IL-6 in ovariectomized and male mice, as well as E2-induced IL-6 in splenocytes from naïve females, suggest critical interactions between E2 and IL-6. We cannot ignore the fact that E2 levels are similar between females, ovariectomized females and males in Fig. 13A (upper panel). However, serum E2 levels at the conclusion of the experiment may not reflect the levels during the experiment. It is possible that our sample may not have coincided with the female mouse estrus cycle [43]. Even so, ovariectomy is an accepted method of diminishing E2 in mice [44].

Prior studies have suggested that high E2 induces Th2 responses [45]. Our studies support this finding by showing that TFA IgG1 antibodies are significantly elevated in the sera from intact females when compared to the levels in sera from ovariectomized female and male mice. We also show that E2 and TFA IgG antibodies are significantly higher in castrated than in non-castrated mice.

In our studies we did not directly address the source of IL-10 in male mice. However, a prior study has shown that testosterone acts directly on CD4+ T cells to increase IL-10 production [15]. An increase in IL-10 could also promote sex bias by encouraging Treg survival in male mice. Hence, in castrated male mice with significantly lower testosterone levels, elevated estrogen and the associated increased IL-6 levels may have contributed to increased experimental DILI. Additionally, estrogen-induced elevated IL-1β levels (Fig. 6A) may have also contributed to increased experimental DILI in castrated males.

We have shown previously that IL-4 initiates immune responses in our model [21]; however, to our knowledge, an association between E2 and IL-6 has not been previously reported in immune-mediated DILI in BALB/c mice. We also show that IL-6 decreases the number of Tregs in both sexes, further supporting our contention that Tregs are not functionally different. This critical finding allows the therapeutic option of Treg supplementation in our model.

We have shown sex bias in splenic but not hepatic Treg levels. This finding may be significant because the spleen is the major extra-hepatic lymphoid organ where critical immune responses occur. Hepatic antigen-presenting cells, such as sinusoidal endothelial cells (LSEC), Kupffer cells (KC), and hepatocytes, weakly induce Treg expansion [46]. Moreover, in the presence of IL-17, LSEC, KC, and hepatocytes overcome Treg suppression [46]. Our IL-17 data may support this idea; however, mechanisms of overcoming hepatic Treg suppression need further investigation. Notably, Treg adoptive transfer lowered hepatic IL-17 and IL-6 levels, suggesting that treatment may hold promise in some cases.

In recent studies, Tregs have been shown to promote tumor survival by promoting tolerance to tumor expansion [47]. Not surprisingly, several of these tumors, namely breast [47], [48], cervical [49], and ovarian [50] cancers, preferentially affect women. This added role for Tregs underscores the delicate balance in critical immune responses that affect cancer and autoimmunity and emphasizes the need for more studies to examine these mechanisms [51].

In conclusion, as we have seen in patients with anesthetic DILI, female BALB/c mice develop more severe experimental DILI from drug haptens than do male mice. Our data suggest that the impairment of splenic Treg expansion by E2-promoted induction of IL-6 and other pro-inflammatory cytokines, as well as E2-promoted enhancement of immune responses to anesthetic DILI antigens may be one mechanism responsible for this susceptibility. Our data show that modulating Treg numbers may provide a means of managing DILI. Similar therapeutic options may ameliorate sex bias in patients who develop immune-mediated DILI after exposure to anesthetics, antibiotics, tienilic acid, dihydralazine, carbamazepine or alcohol.
